# Supplementary material for: A Mycobacterium tuberculosis rare variable antigen vaccine reduces lung pathology in hypersusceptible SP140−/− mice
Source: Front Tuberc. 2026 Jul 15;4:1814646. doi: 10.3389/ftubr.2026.1814646 (PMC13491433; doi:10.3389/ftubr.2026.1814646)
Supplement: Supplementary file 1 [file Datasheet1.docx]

**Supplementary Material**

**Supplementary Figure 1. Gating strategy for assessing CD4 T cell subsets**. For cytokine and transcription factor staining, only the CD4^+^ T cell CD44^+^ population was assessed.

**Supplementary Figure 2. Epitope mapping for RimJ using pooled overlapping peptides in spleen and mediastinal lymph node.** Pooled overlapping peptides (18-mers overlapping by 11) spanning the length of the RimJ antigen were used in small (2-4 peptides) pools at a concentration of 10 μg/ml to stimulate cells from spleen and lymph nodes from Mtb H37Rv-infected C57BL/6 mice *ex vivo* for 72 hours. **(A)** Spleen cell responses as measured by IFNγ concentration in the cell culture supernatant revealed a dominant response to two peptides spanning amino acids 92-116. Data is mean±SEM (n = 3 mice). **(B-C)** Mediastinal lymph nodes from 5 mice infected with Mtb Erdman were pooled at 21 days post-infection. IFNγ and IL-17 concentrations in the cell culture supernatant revealed responses targeted towards amino acids 92-116 and 172-203. Graphs represent one experiment with one sample per 3 peptide pool. **(D)** Peptide binding affinity for the H2-IAb allele was predicted using IEDB NetMHCIIpan 4.0 BA using the RimJ sequence and 15-18 amino acid peptide length. Predicted binding affinity ranked by percentile ranking was graphed for the best predicted binding peptides for the designated regions of RimJ. The prediction revealed similar hotspots to the regions identified in the stimulation experiments **(A-C)**. Labeled rectangles (99-116 and 182-197) are the peptides predicted to stimulate the majority of the response to RimJ-Erdman.

**Supplementary Figure 3. IL-17A response to RimJ in the mediastinal lymph node and IL-17A reporter expression in CD4 T cells during Mtb infection**. **(A)** C57BL/6 mice were infected with Mtb Erdman, and mediastinal lymph nodes were pooled from 5 mice at each timepoint and cultured in triplicate in the presence of ESAT-6 peptides (10 μg/ml), RimJ peptides (10 μg/ml), or no peptides for 72 hours. IFNγ and IL-17A were measured in the culture supernatants by ELISA. **(B)** Lung cells from C57BL/6 mice infected with Mtb Erdman 28 days prior, were stimulated with Mtb whole cell lysate (10 μg/ml), ESAT-6 peptides (10 μg/ml), or RimJ peptides (10 μg/ml) for ICS. Frequencies of IFNγ^+^ or IL-17A^+^ CD4 T cells were determined by flow cytometry. **(C-D)** *Il17a-cre*; Ai14 reporter mice were infected with Mtb Erdman and used to identify IL-17A-producing cells by fate-mapping. **(C)** Transcription factor staining of lung cells from infected reporter mice. **(D)** Stimulation of lung cells for ICS to identify IFNγ^+^ and IL-17A^+^ cells.

**Supplementary Figure 4. Analysis of lung immunofluorescence images.** **(A)** Representative images depicting the manual assignment of the core and cuff regions of the identified lesions. **(B)** Masking of the positive signal for each marker using pixel thresholders in QuPath to create objects for CD4^+^ and CD68^+^ cells and measure total area for Ly6G, α-SMA, E-cadherin, and Mtb (ab905) staining. **(C)** Dual channel images showing α-SMA (green), E-cadherin (red), and Mtb (ab905, orange) with DAPI counterstain from a representative pCas4-vaccinated mouse lesion.

**Supplementary Figure 5. B cell numbers in SP140^-/-^ mice after anti-CD20 B cell depletion during vaccination**. **(A)** Experimental timeline showing mice vaccinated with mock (Empty Vector) or pCas4 (Cassette 4) and treated with anti-CD20 antibody or not. (**B)** B cells (CD19+) are nearly completely depleted from the blood of anti-CD20 (αCD20) treated mice at 7 days post-depletion, as compared to mice that did not receive antibody (Control). The frequency **(C)** and total number **(D)** of parenchymal B cells (CD19^+^ cells) at 21 days post-infection was slightly reduced in mice treated with anti-CD20 antibody, but not significantly different (ANOVA).
